# Changes in the spatial distribution of the Purkinje network after acute myocardial infarction in the pig

**DOI:** 10.1371/journal.pone.0212096

**Published:** 2019-02-11

**Authors:** Victor Garcia-Bustos, Rafael Sebastian, Maite Izquierdo, César Rios-Navarro, Vicente Bodí, Francisco Javier Chorro, Amparo Ruiz-Sauri

**Affiliations:** 1 Department of Pathology, Faculty of Medicine, Universitat de Valencia, Valencia, Spain; 2 Department of Internal Medicine Unit, Hospital Universitari i Politècnic La Fe, Valencia, Spain; 3 Computational Multiscale Simulation Lab, Universitat de Valencia, Valencia, Spain; 4 INCLIVA Biomedical Research Institute, Valencia, Spain; 5 Department of Cardiology, Hospital Clínico Universitario de Valencia, Valencia, Spain; 6 CIBERCV, Carlos III Health Institute, Madrid, Spain; University of Minnesota, UNITED STATES

## Abstract

Purkinje cells (PCs) are more resistant to ischemia than myocardial cells, and are suspected to participate in ventricular arrhythmias following myocardial infarction (MI). Histological studies afford little evidence on the behavior and adaptation of PCs in the different stages of MI, especially in the chronic stage, and no quantitative data have been reported to date beyond subjective qualitative depictions. The present study uses a porcine model to present the first quantitative analysis of the distal cardiac conduction system and the first reported change in the spatial distribution of PCs in three representative stages of MI: an acute model both with and without reperfusion; a subacute model one week after reperfusion; and a chronic model one month after reperfusion. Purkinje cells are able to survive after 90 minutes of ischemia and subsequent reperfusion to a greater extent than cardiomyocytes. A decrease is observed in the number of PCs, which suffer reversible subcellular alterations such as cytoplasm vacuolization, together with redistribution from the mesocardium—the main localization of PCs in the heart of ungulate species—towards the endocardium and perivascular epicardial areas. However, these changes mainly occur during the first week after ischemia and reperfusion, and are maintained in the chronic stages. This anatomical substrate can explain the effectiveness of endo-epicardial catheter ablation of monomorphic ventricular tachycardias in the chronic scar after infarction, and sets a basis for further electrophysiological and molecular studies, and future therapeutic strategies.

## Introduction

Patients with ischemic heart disease are at an increased risk of suffering sustained ventricular tachycardia (VT). Sustained monomorphic VT occurs most frequently in the chronic stage of myocardial infarction (MI). Re-entry is the mechanism underlying monomorphic VT. Such re-entry is sustained by regions of surviving myocardial cells (cardiomyocytes and Purkinje cells (PCs), among others) that conform channels within the fibrous scar. In this setting, PCs are known to be more resistant to ischemia than myocardial cells, as evidenced in canine hearts [1]. Purkinje cell arrhythmogenic mechanisms are well known and intervene in reentry circuits in the infarction scar [2].

Many factors have been shown to increase the risk of ventricular tachycardia late after MI, including a large scar size, due to the lack of early revascularization. However, structural remodeling over time may determine the arrhythmogenic substrate and risk. Added to all these considerations, and although the location of the arrhythmogenic substrate in chronic infarction is said to be mainly in the endocardium, many ablation studies have identified frequent epicardial and mid-myocardial substrates [3].

Although there is abundant information on the electrical properties and histological subcellular alterations of PCs after myocardial infarction [1, 4–11], most of the published studies only explore the acute phase of MI in just one type of infarction model from the perspective of reperfusion. Furthermore, there are no descriptions referred to the exact location of these PCs in the scar. The aim of the present study was to compare the histological changes in PCs over time and in different post-infarction stages—acute, subacute and chronic—as well as in reperfused versus non-reperfused infarction.

## Materials and methods

### Experimental procedure

Twenty-four juvenile adult domestic pigs weighing 25–30 kg were included in the study. The experimental procedure was approved by the Animal Care and Use Ethics Committees of the University of Valencia and the INCLIVA Biomedical Research Institute, and complies with the Guide for the Care and Use of Laboratory Animals published by the United States National Institutes of Health (NIH Publication No. 85–23, revised 1996) and with the ARRIVE guidelines (S1 File).

The study protocol was designed and conducted as previously described [12]. Intramuscular ketamine (8 mg/kg) and medetomidine (0.1 mg/kg) were used for sedation, and a continuous intravenous infusion of 2% propofol (10 mg/kg/h) was used as anesthesia. Pigs were monitored by continuous electrocardiography (ECG) and were mechanically ventilated with 50% oxygen. To prevent life-threatening arrhythmias and thrombosis, the animals were pre-treated with intravenous amiodarone (150 mg), lidocaine (30 mg) and heparin (3000 IU).

A 7F sheath was inserted into the right femoral artery to monitor blood pressure and access the left anterior descending coronary artery (LAD), through which a 7F Amplatz Left 0.75 catheter was selectively positioned. A standard hydrophilic angioplasty wire was placed in the distal LAD and an over-the-wire 2.5 x 15 mm angioplasty balloon was inflated at 6 atm in the mid-LAD segment distal to the first diagonal branch in order to provoke acute MI. Total coronary artery occlusion was confirmed by ST-segment elevation on the ECG and angiography.

Five experimental groups were established: control (n = 2), acute non-reperfused infarction (n = 4), acute reperfused infarction (n = 5), subacute reperfused infarction (n = 9) and chronic reperfused infarction (n = 4). In the control group, the same procedure was performed, but the angioplasty balloon was not inflated. Under general anesthesia and unconsciousness, the hearts were arrested with potassium chloride and histological preparation was carried out immediately after explantation. In the acute non-reperfused infarction group the balloon was not deflated and the heart was arrested and removed after 90 minutes of ischemia. In the acute reperfused infarction, subacute reperfused infarction and chronic reperfused infarction groups, the balloon was deflated after 90 minutes of ischemia and coronary flow was respectively restored for one minute, one week and one month, respectively.

### Tissue sampling and processing

**Fig 1:**
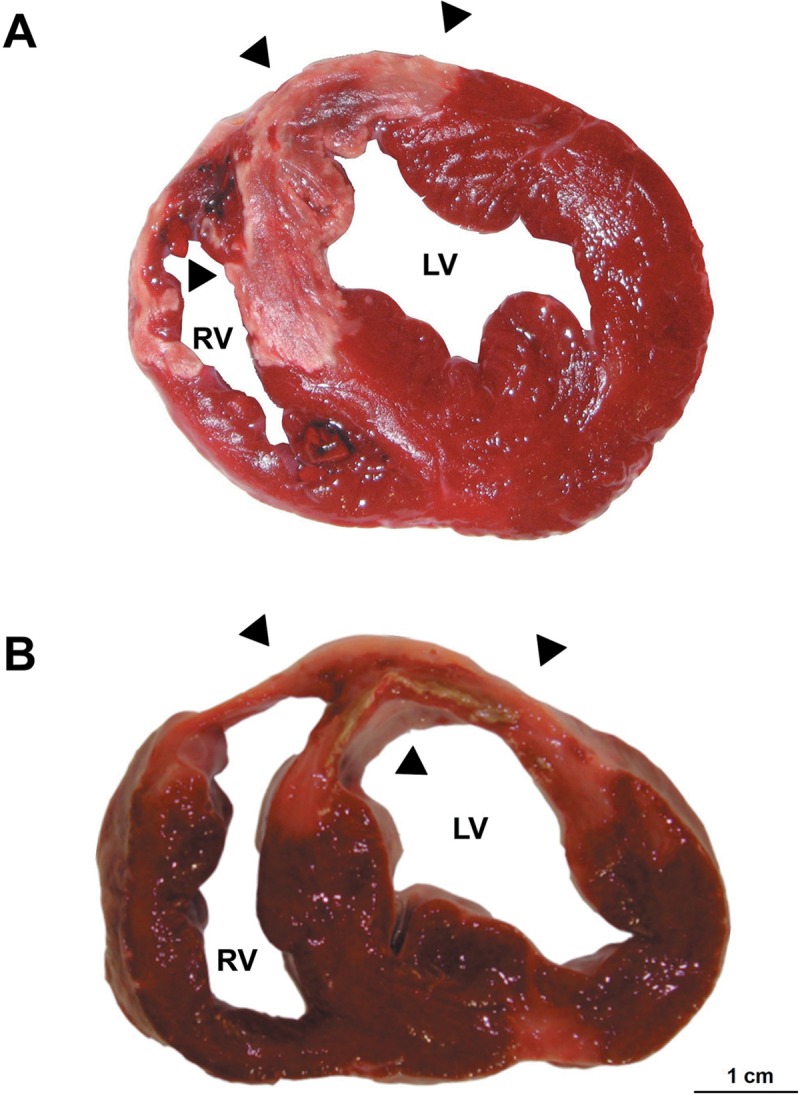
Macroscopic section of the pig heart after acute myocardial infarction, stained with 2,3,5-triphenyltetrazolium chloride. A. Sample from the subacute reperfused infarction group. B. Sample from the chronic reperfused infarction group. Arrows indicate the area of infarction (not stained with 2,3,5-triphenyltetrazolium chloride). Note the transmural nature of the infarction zone, which appears well defined and paler. LV, left ventricle; RV, right ventricle.

Each left ventricle was dissected and sectioned into 5 mm-thick short axis slices, followed by incubation in a 2% 2,3,5-triphenyltetrazolium chloride solution (TTZ; Merck Millipore, Billerica, MA, USA) for 20 minutes at 37°C to identify the infarcted myocardium, which was defined as the area that did not stain with TTZ and was ensured to be transmural (Fig 1). All samples were taken from the core infarcted area. Samples were tagged for identification, fixed in 4% formaldehyde and embedded in paraffin. Histological sections measuring 5 μm in thickness were obtained with a microtome and subjected to staining with hematoxylin-eosin (HE).

In the control group, several transmural samples were taken from the anterior myocardial area irrigated by the anterior descending coronary artery.

### Histopathological evaluation and imaging analysis

The histological evaluation and imaging analysis were performed with a Leica DMD108 optic light microscope (Leica Microsystems, Wetzlar, Germany), and the overall distribution of the Purkinje fibers of the tissue was mapped by acquiring high-quality low magnification photographs in order to ensure every Purkinje cell was localized in the sample. We compiled 800 photomicrographs at different magnifications (40x, 100x, 200x, 400x and 630x) including Purkinje fibers. Identification of PCs was based on their larger size without clearly defined intercalated disks^17^ and differential staining features. Each PC was captured in our studied tissue in order to minimize the inaccuracy of average measures in our model attributable to their natural heterogeneity and possible alteration of their distribution induced by prolonged ischemia. The computerized morphometric study was carried out using biomedical imaging analysis software (Image Pro-Plus 7.0) (Media Cybernetics, Silver Spring, MD, USA). From the complete sample set, we obtained 300 micrographs from a total of 27 slides in order to perform an exhaustive analysis.

We defined several major morphometric variables, as described elsewhere [13]:

- Purkinje density under 100x magnification, expressed as the percentage tissue area covered by PCs in the micrographs: Firstly, each micrograph was RGB color system segmented, and the green channel was chosen and calibrated. The total tissue area (in μm^2^) was recorded after its manual chromatic histogram-based selection. The mean raw tissue area of the captions was 1.62 mm^2^. Using the original hematoxylin-eosin calibrated micrograph, the PCs were manually measured and the density corresponding to Purkinje fibers was calculated as the percentage of the total tissue area occupied by them in each micrograph. Thick and thin bundles of PCs, terminal fibers and Purkinje-cardiomyocyte junctions (PMJs) were included in the analysis. Transitional T fibers were not considered in the measurements.
- Purkinje cell vacuolization: A semi-quantitative measurement of PC vacuolization was carried out. Each photomicrograph was defined as being vacuolated if 50% or more of the PCs were vacuolated in 50% or more of their cytoplasmic component.
- Maximum thickness of PC bundles: Several measures of the thickest portion of each bundle were taken manually under 100x magnification in the transversal and longitudinal central sections of the Purkinje bundles. The selection included both the Purkinje fibers and the surrounding connective tissue. The mean of each micrograph was then calculated.
- Purkinje-cardiomyocyte junctions (PMJs): These were represented adopting a dichotomic approach as the presence or absence of optically detected PMJs per micrograph, together with their density per mm^2^, by dividing the total PMJ count of each group by the sum of the areas of each group in mm^2^. The type of PMJ was also determined as previously established: contact through cell body (CCB), contact through cell prolongations (CCP) and contact through transitional cells (CTC).
- Localization: Each PC measurement was also classified according to cardiac distribution, namely subendocardial, intramyocardial and epicardial, and the perivascular distribution was also recorded if present. Those PCs which were closely related to vessels (around 150 μm) and appeared apposed to the vascular connective tissue were considered to have a perivascular location.

### Statistical analysis

Descriptive statistics, graphic representations and contrasting of hypotheses were performed with the R-3.3.4 package (R Core Team, Vienna, Austria). Continuous variables were expressed as the mean and standard deviation, while categorical variables were expressed as the percentage and 95% confidence interval (95%CI). Statistical significance was considered for p<0.05. Normal data distribution was assessed from quantile-quantile plots and the Shapiro-Wilk test. All continuous variables exhibited a non-normal distribution; the nonparametric Kruskal-Wallis test was therefore chosen chosen, and pairwise comparisons were carried out using the Steel-Dwass p-adjustment method. The Fisher exact test was used to analyze qualitative variables. When the expected count of any level was less than 5, correction was applied by means of a Pearson's chi-squared test with Monte-Carlo simulated p-value (based on 2000 replicates).

All tissue processing, histopathological evaluations, data collection and statistical analyses were blinded.

## Results

### 1. Histopathological description of Purkinje cells

**Fig 2:**
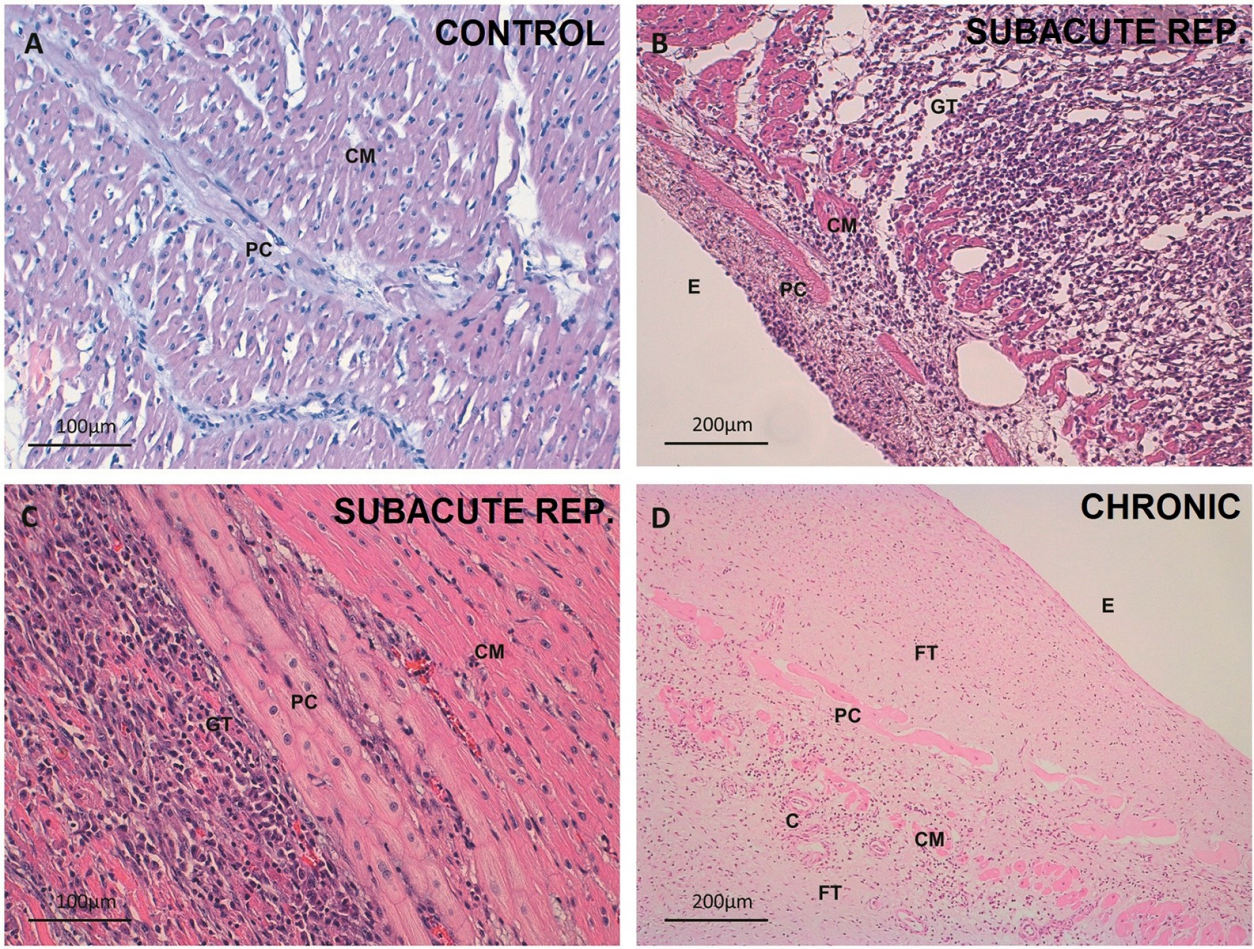
Tissue changes in the different spatial distributions of Purkinje cardiomyocytes after myocardial infarction. A. Healthy intramyocardial Purkinje cells. Control group. 200x magnification. Hematoxylin-eosin. B. Subendocardial layer of Purkinje fibers in the subacute reperfused infarction group. 100x magnification. Hematoxylin-eosin. Practically all the myocardial tissue has been replaced by mixed granulation and inflammatory tissue. Purkinje cells appear only slightly vacuolated, before a layer of disrupted surviving cardiomyocytes. Inflammatory infiltrate can also be seen in the endocardium. C. Detail of an unaltered intramyocardial Purkinje bundle delimitating an area of necrotic tissue and inflammatory infiltrate in preserved myocardium in the subacute reperfused infarction group. 200x magnification. Hematoxylin-eosin. D. Preserved subendocardial Purkinje cells within an extensive fibrous tissue scar. Chronic infarction group. 100x magnification. Hematoxylin-eosin. Note the presence of several cardiomyocytes as well as the appearance of capillaries. C, capillary; CM, cardiomyocyte; E, endocardium; FT, fibrous tissue; GT, granulation tissue; PC, Purkinje cell.

**Fig 3:**
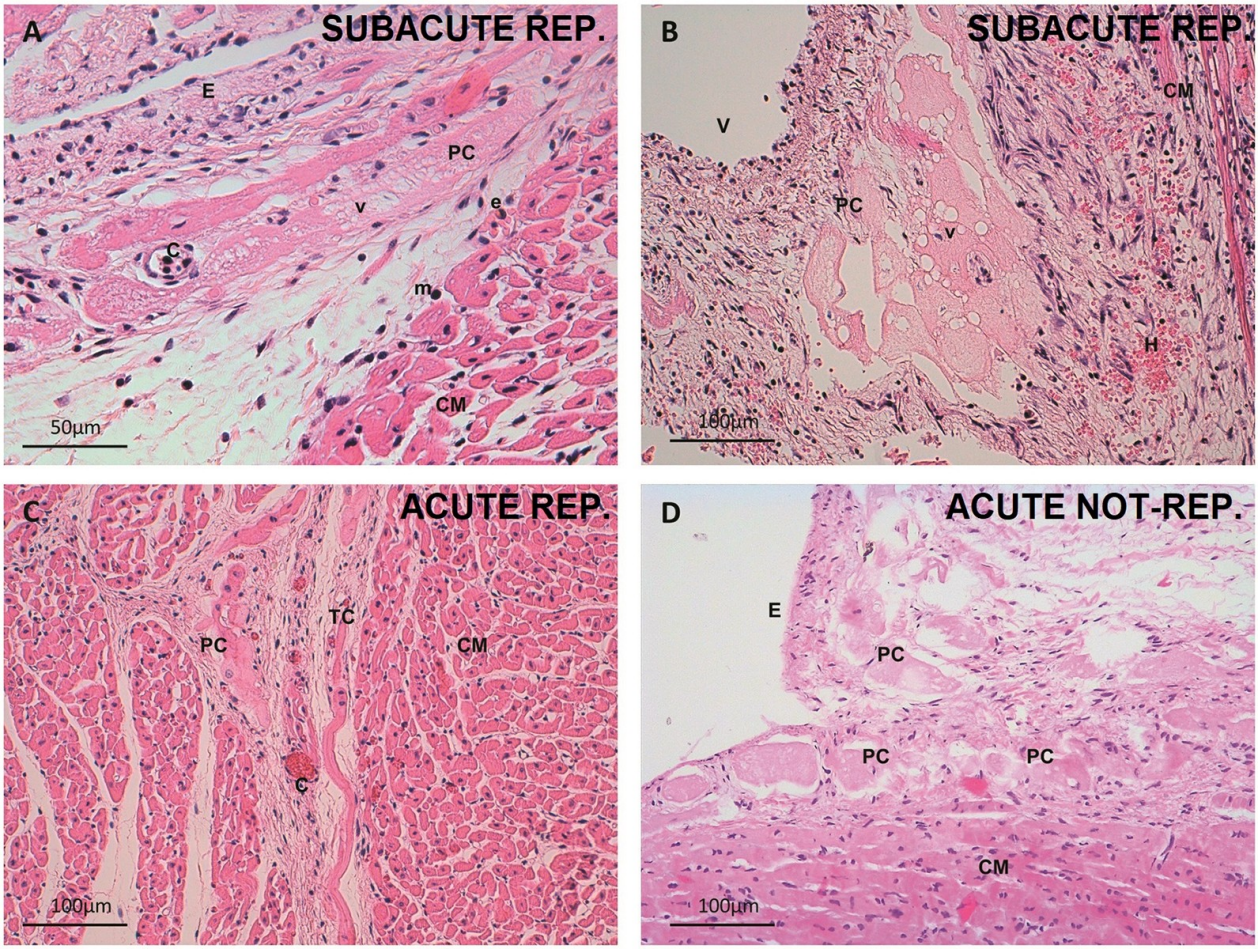
Cellular changes in Purkinje cardiomyocytes after myocardial infarction. A. Detail of a vacuolated subendocardial Purkinje cell. Subacute reperfused infarction group. 400x magnification. Hematoxylin-eosin. Microvacuoles predominate in this micrograph. B. Cluster of perivascular Purkinje cells with large vacuoles. Subacute reperfused infarction group. 200x magnification. Hematoxylin-eosin. An active fibroblastic response and hemorrhage can be seen. C. Intramyocardial Purkinje and transitional cells in the acute phase. Acute reperfused infarction group. 200x magnification. Hematoxylin-eosin. Note microvascular obstruction of the capillaries. D. Necrotic subendocardial Purkinje cells and cardiomyocytes in the acute non-reperfused infarction group. 200x magnification. Hematoxylin-eosin. C, capillary; CM, cardiomyocyte; E, endocardium; e, eosinophil; m, mononuclear cell; TC, transitional cell; PC, Purkinje cell; V, vein; v, vacuoles.

**Fig 4:**
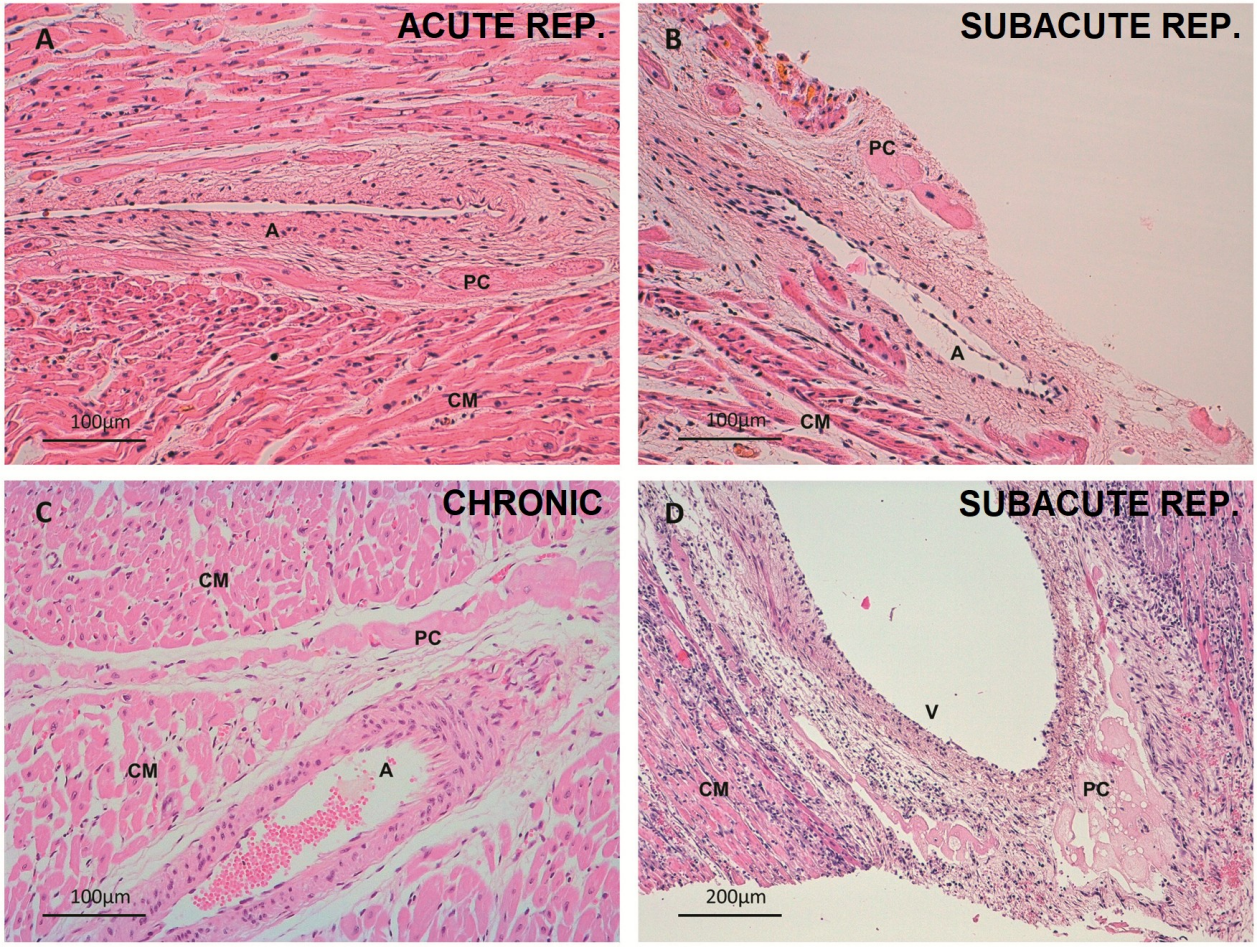
Perivascular Purkinje cardiomyocytes. A. Intramyocardial perivascular Purkinje cells. Acute reperfused infarction group. 200x magnification. Hematoxylin-eosin. Note acute hypereosinophilia of inferior cardiomyocytes. B. Small epicardial cluster of pericoronary Purkinje cells. Subacute reperfused infarction group. 200x magnification. Hematoxylin-eosin. C. Perivascular penetrating Purkinje cells. Chronic infarction group. 200x magnification. Hematoxylin-eosin. D. Cluster of perivascular vacuolated Purkinje cells. Subacute reperfused infarction. 100x magnification. Hematoxylin-eosin. Tissue necrosis and inflammatory infiltrate are evident. A, artery; CM, cardiomyocyte; PC, Purkinje cell; V, vein.

In the control group, the PCs were larger and paler in appearance than the cardiomyocytes. The PCs were organized both in a subendocardial network and within the myocardium in the form of thick penetrating bundles coexisting with thin terminal Purkinje fibers and their PMJs (Fig 2A). In the acute reperfused infarction samples, no subjective structural changes were observed, and cytoplasmic vacuolization was already present in several PCs (Fig 3). Some of these cells were observed in the perivascular space. In the acute non-reperfused infarction group, the subjective findings resembled those recorded in the acute reperfused infarction samples. In the subacute reperfused infarction group, the nuclei were preserved, but the cytoplasm contained abundant micro- and macrovacuoles (Fig 3B). A substantial number of PCs exhibited a perivascular distribution, and some were observed in the epicardium (Fig 4B and 4D). The chronic reperfused samples showed a considerable loss of PCs. The cells were more frequently seen in the subendocardium, and occasional vacuolization or karyorrhexis was seen. Some resistant PCs were not structurally altered and sometimes became the only visible cells within the collagen matrix (Fig 2D).

The tissue and cell characteristics, as well as the features of the perivascular PCs can be seen in further detail in Figs 2–4.

### 2. Purkinje cell density, vacuolization and distribution

#### Purkinje cell density

**Fig 5:**
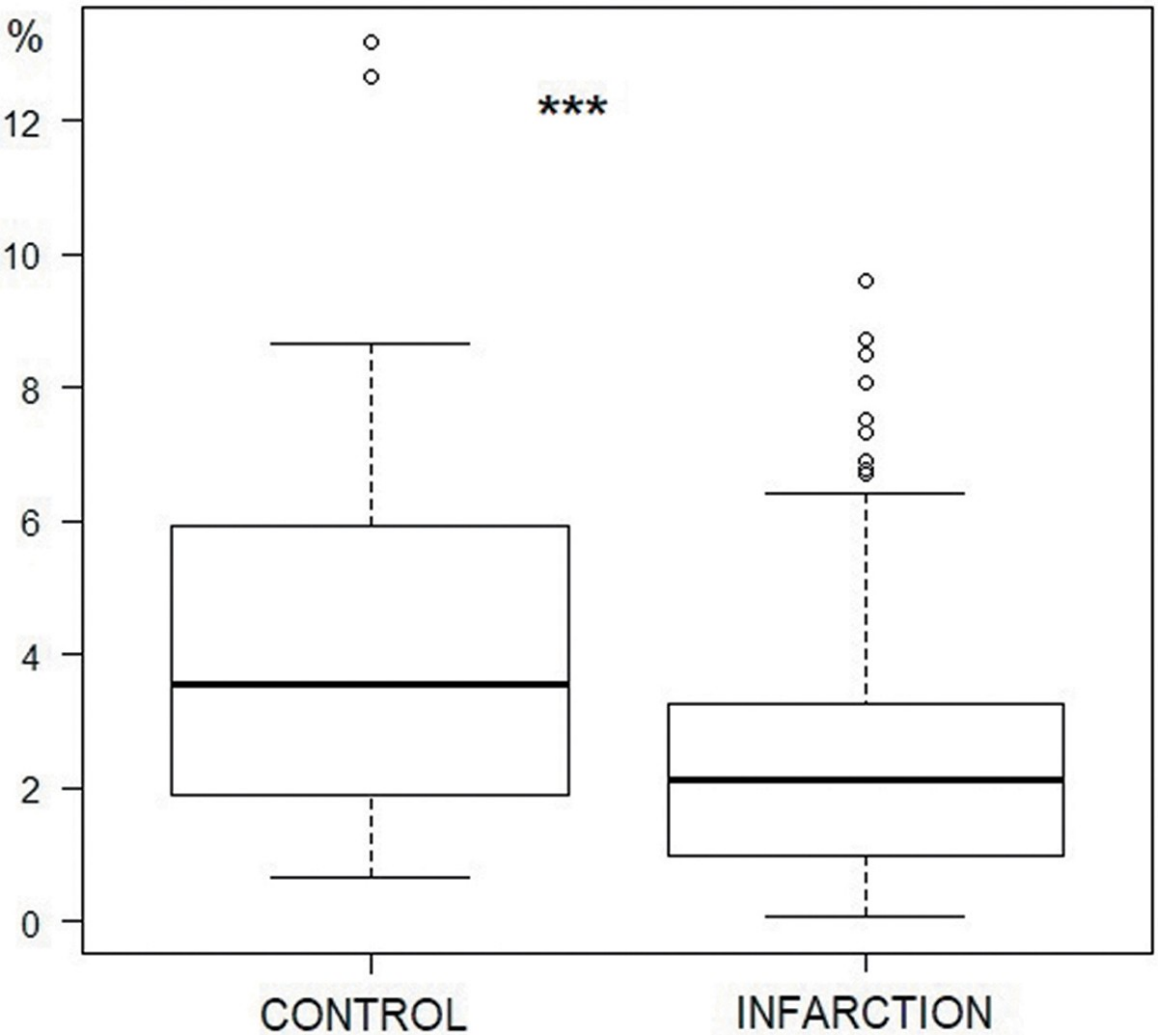
Density of Purkinje fibers in the control and infarction groups. Density is expressed as a percentage of the total area of the tissue (***p<0.001, Fisher exact test).

More than 300 micrographs were assessed. The mean PC density was significantly lower in samples of infarcted myocardium than in healthy tissue (4.21% vs 2.42%; p<0.01) (Fig 5).

**Fig 6:**
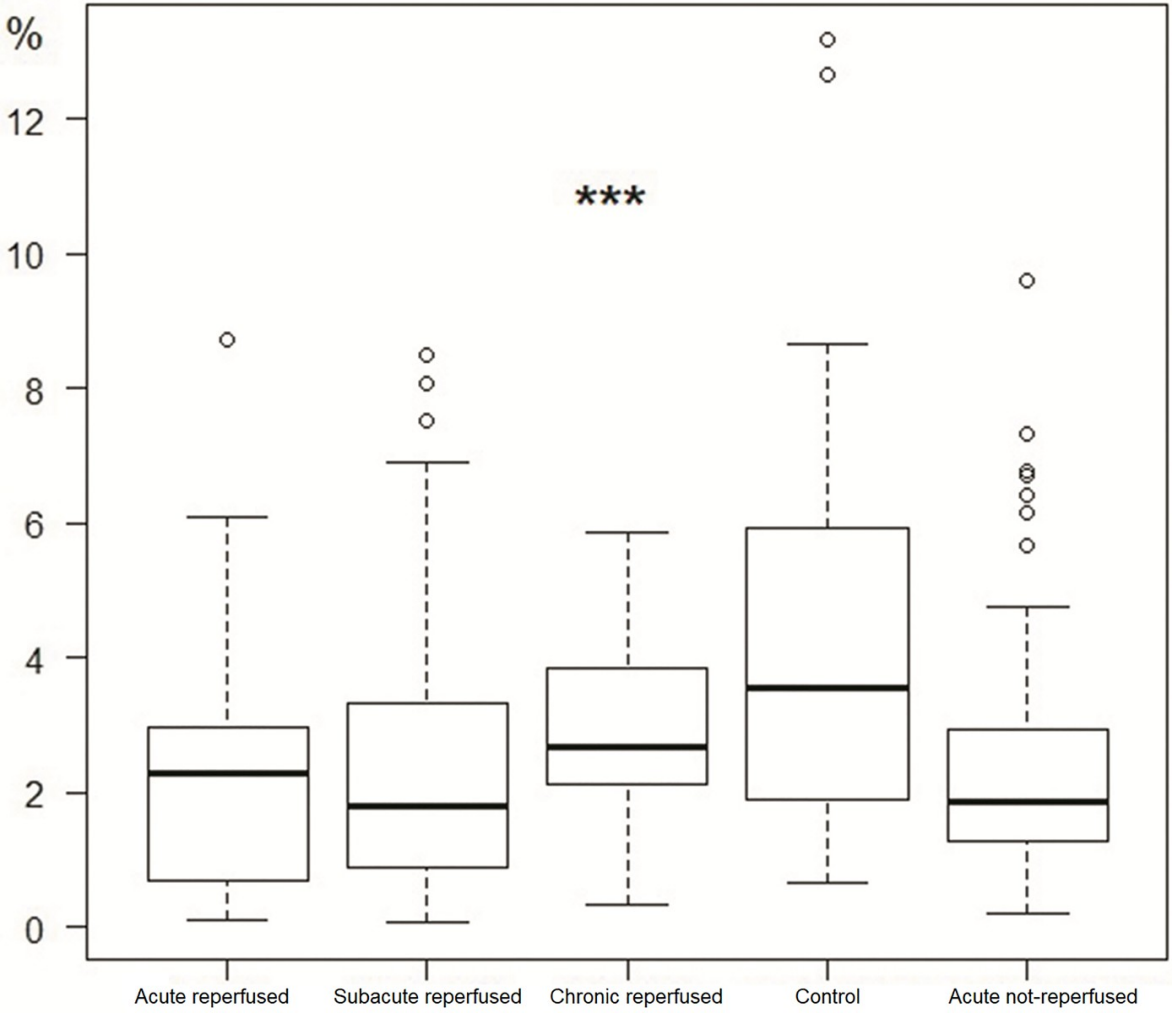
Density of Purkinje fibers in the acute reperfused infarction, subacute reperfused infarction, chronic reperfused infarction, control and acute non-reperfused infarction groups (***p<0.001, Fisher exact test).

A small but significant decrease in PC density was found in the acute reperfused infarction, acute non-reperfused infarction and subacute reperfused infarction groups compared with the control group (see Table 1 and Fig 6). The absolute PC density in the chronic reperfused infarction group was higher than in the controls, as the proportional loss of myocardial tissue was greater than the loss of PCs. The difference was not statistically significant, however.

**Table 1:** Summary of the main morphometric parameters of the Purkinje cardiomyocytes in all the experimental groups.

Parameter	Control	Acute non-reperfused infarction	Acute reperfused infarction	Sub-acute reperfused infarction	Chronic reperfused infarction	p-value
**PMJ (%, CI)**	78.6 (65.6, 88.4)	52.6 (40.8, 64.2)	48.8 (32.9, 64.9)	49 (38.9, 59.2)	33.3 (15.6, 55.3)	* p<0.0001
**Perivascular distribution (%, CI)**	0 (0, 5.2)	4.1 (0.8, 11.4)	2.5 (0.1, 13.2)	20.2 (12.8, 29.5)	16.7 (4.7, 37.4)	* p<0.0001
**Max. thickness Mean (μm, SD)**	144.21 (53.26)	99.37 (58.78)	76.30 (37.52)	72.29 (39.56)	129.25 (81.78)	^*†*^ p<0.0001
**PC density (%, SD)**	2.8 (1.46)	2.41 (1.83)	2.33 (1.99)	2.38 (1.93)	4.21 (2.87)	* p<0.0001
**Vacuolization (%, CI)**	7 (1.9, 17)	23.3 (14.2, 34.6)	36.6 (22.1, 53.1)	43.3 (32.9, 54.2)	25 (9.8, 46.7)	* p<0.0001

CI, confidence interval; PMJ, Purkinje-myocardial junction; PC, Purkinje cardiomyocyte.

*Fisher exact test.

† Kruskal-Wallis test.

#### Purkinje cell vacuolization

Differences in the percentage of photomicrographs showing abundant PC vacuolization were also seen. In the control group, less than 10% of the photomicrographs evidenced PC vacuolization. The latter was significantly increased in all the experimental groups with respect to the controls (Table 1). An increasing tendency in cytoplasmic vacuolization rate was observed from the acute to the subacute phases of infarction, with the rate being lower in the chronic stage. The absolute vacuolization rate was lower in the non-reperfused infarction group, but failed to reach statistical significance.

#### Purkinje cell bundle thickness

**Fig 7:**
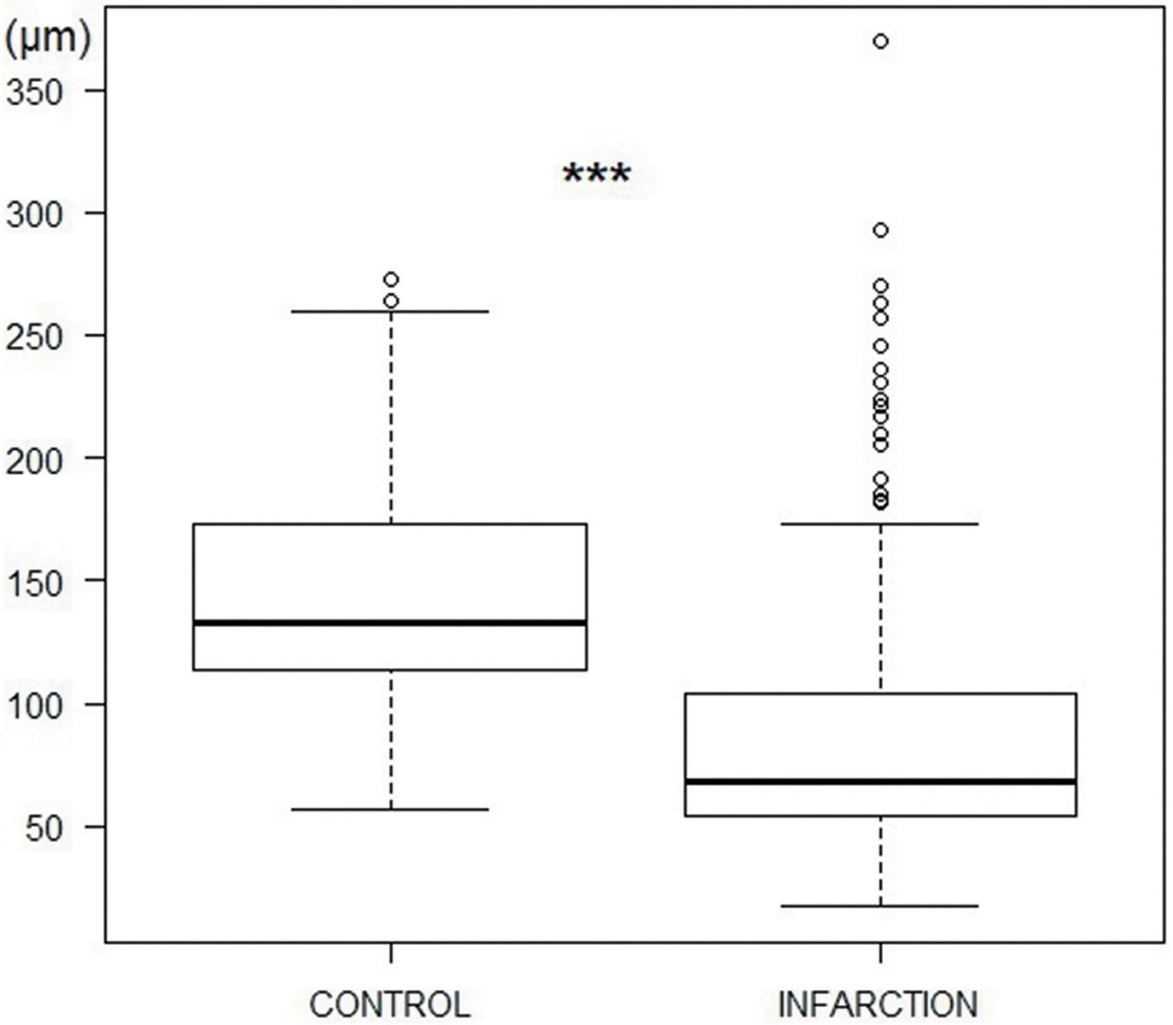
Purkinje bundle thickness (μm) in the control and infarction groups (***p<0.001, Kruskal-Wallis test).

**Fig 8:**
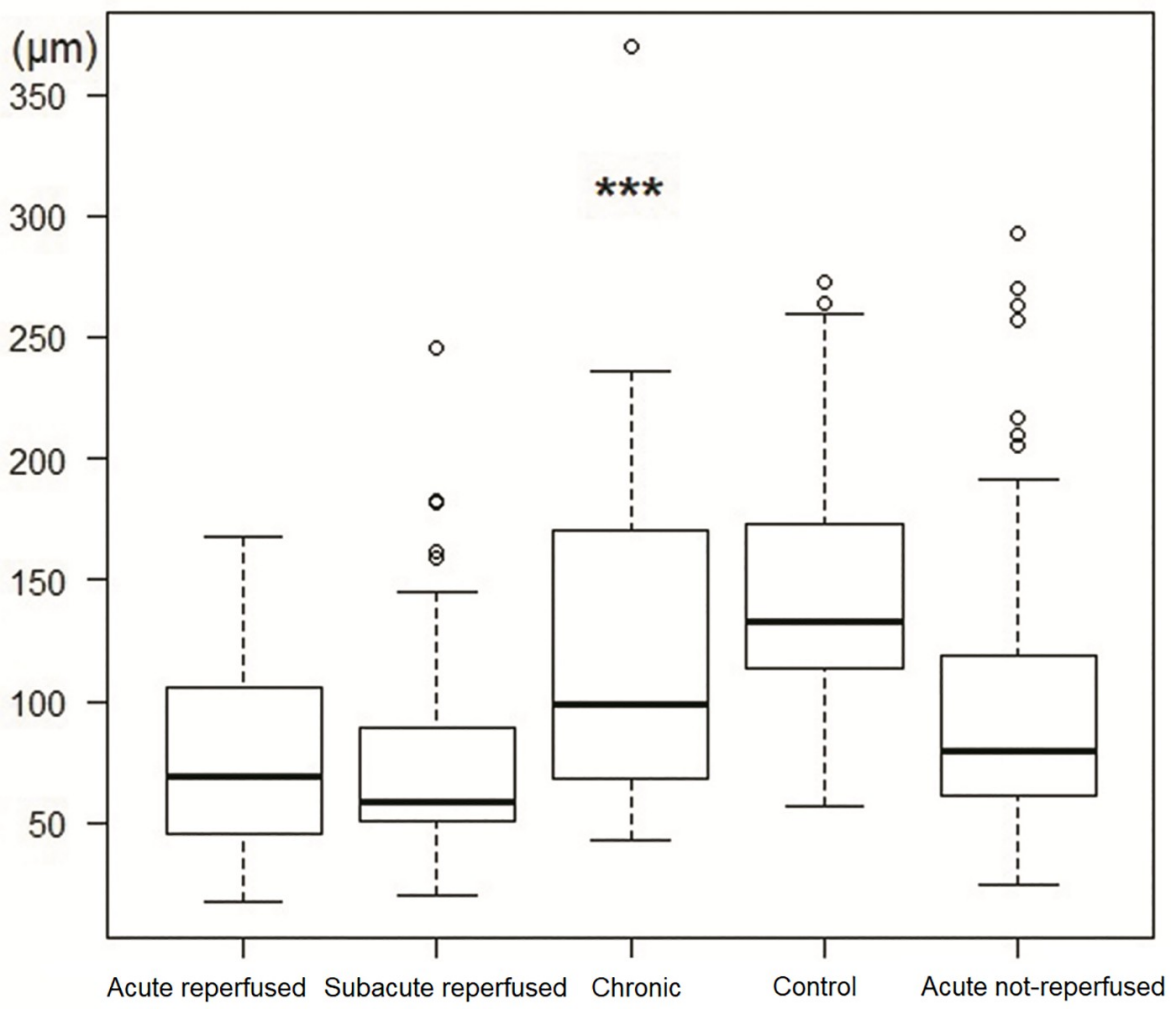
Purkinje bundle thickness in the acute reperfused infarction, subacute reperfused infarction, chronic reperfused infarction, control and acute non-reperfused groups (***p<0.001, Kruskal-Wallis test).

Globally, the PC bundles significantly decreased their thickness in the infarction samples in comparison to the control group (144.2±53.3 vs 87.3±54.2 μm; p<0.001) (Fig 7). On analyzing the experimental groups, the acute reperfused infarction, subacute reperfused infarction and acute non-reperfused infarction groups showed thinner PC bundles than the control group (Table 1, Fig 8). In the chronic reperfused infarction group, a tendency towards thinner PC bundles than in the control group was observed, though the difference was not statistically significant.

#### Purkinje-cardiomyocyte junctions

A loss of PMJs was found in all the infarction stages (reperfused and non-reperfused) in comparison with the control group (78.6% (65.6–88.4) vs 48.5% (42–55); p<0.01)(Table 2).

**Table 2:** Frequency of the different patterns of Purkinje-cardiomyocyte junctions in the control and infarction groups.

Type of PMJ	Control	Infarction
**CCB (%, CI)**	57.8 (42.2, 72.3)	49.6 (40.2, 59)
**CCP (%, CI)**	35.6 (21.9, 51.2)	30.8 (22.6, 40)
**CTC (%, CI)**	6.7 (1.4, 18.3)	19.7 (12.9, 28)

p = 0.127 (Fisher test). Data are represented as percentage and 95% confidence interval. CCB, contact through the cell body; CCP contact through cell prolongations; CI, confidence interval; CTC contact through transitional cells; PMJ, Purkinje-cardiomyocyte junction.

**Fig 9:**
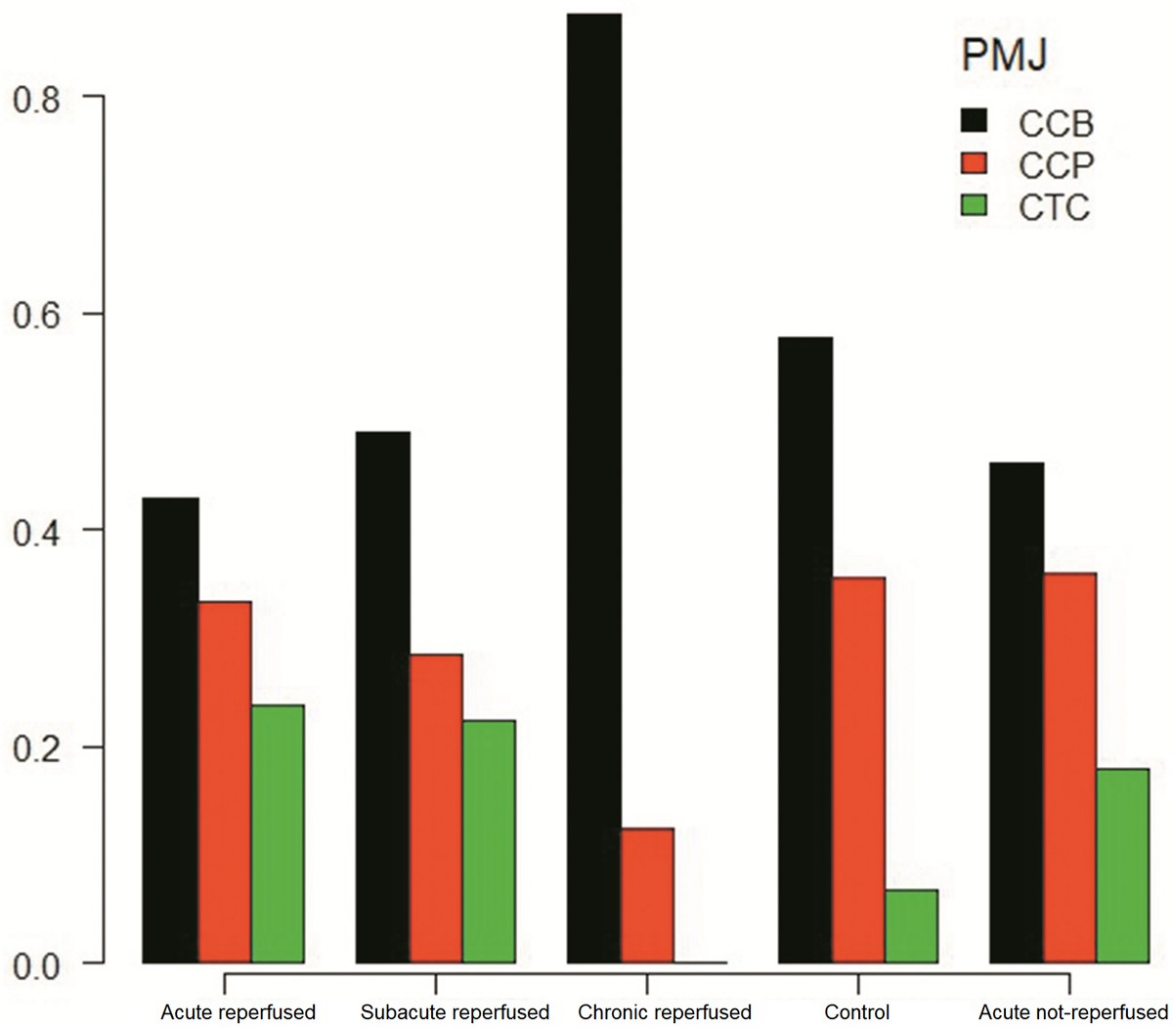
Frequency of the different types of PMJs in the acute reperfused infarction, subacute reperfused infarction, chronic reperfused infarction, control and acute non-reperfused groups (p = 0.23, Pearson's chi-squared test with simulated p-value (based on 2000 replicates)). CCB, contact through the cell body; CCP, contact through cell prolongations; CTC, contact through transitional cells; PMJ, Purkinje-cardiomyocyte junction.

In the controls, contact through cell bodies (CCB) was the most frequent type of PMJ, followed by contact through cell prolongations (CCP). Contact through transitional cells (CTC) was the least common type of PMJ. This distribution was maintained in all infarcted tissue groups (Table 3, Fig 9). In this respect, a significant decrease in the number of PMJs was observed, but no change in the type of junctions was noted.

**Table 3:** Frequency of the different patterns of Purkinje-cardiomyocyte junctions in all the experimental groups.

Type of PMJ	Control	Acute non-reperfused infarction	Acute reperfused infarction	Subacute reperfused infarction	Chronic reperfused infarction
**CCB (%, CI)**	57.8 (42.2, 72.3)	46.2 (30.1, 62.8)	42.9 (21.8, 66)	49 (34.4, 63.7)	87.5 (47.3, 99.7)
**CCP (%, CI)**	35.6 (21.9, 51.2)	35.9 (21.2, 52.8)	33.3 (14.6, 57)	28.6 (16.6, 43.3)	12.5 (3, 52.7)
**CTC (%, CI)**	6.7 (1.4, 18.3)	17.9 (7.5, 33.5)	23.8 (8.2, 47.2)	22.4 (11.8, 36.6)	0 (0, 31.2)

p = 0.238 (Pearson's chi-squared test with Monte-Carlo simulated p-value (based on 2000 replicates)). CCB, contact through the cell body; CCP, contact through cell prolongations; CI, confidence interval; CTC, contact through transitional cells.

#### Purkinje cell location

We examined 197 micrographs (100x magnification) for the analysis of this variable: 144 from the mid-myocardium, 131 from the subendocardium, and 22 from the epicardium.

In the control group, PCs were more frequently found in the mid-myocardium; the second location in order of frequency was the subendocardium. No epicardial PCs were found. In contrast, in the infarction groups, PC distribution within the three myocardium layers changed significantly. Considering the global infarction samples, a significant loss of mid-myocardial PCs was recorded, with an increase in PCs in the endocardium versus the control group.

In the analysis of the different groups, we observed a progressive decrease in mid-myocardial PCs from the control group to the acute infarction phase and finally the subacute phase. The notorious loss of intramyocardial PCs in the subacute phase of infarction was subsequently maintained in the chronic phase of the reperfused infarction samples (Table 1). This progressive decrease in mid-myocardial PCs in the different infarction stages proved statistically significant. Concurrently, a progressive increase was observed in the number of subendocardial PCs on comparing the control versus the acute reperfused infarction group versus the subacute reperfused infarction group. There were no further differences between the subacute reperfused infarction group and the chronic reperfused infarction group (Table 4).

**Table 4:** Frequency of different localization patterns of Purkinje cardiomyocytes in all the experimental groups.

Localization	Control	Acute non-reperfused infarction	Acute reperfused infarction	Subacute reperfused infarction	Chronic reperfused infarction
**Subendocardial (%, CI)**	23.2 (13, 36.4)	36.8 (26.1, 48.7)	41.5 (0.263, 0.579)	61 (50.7, 70.6)	50 (29.1, 70.9)
**Intramyocardial (%, CI)**	76.8 (63.6, 87)	60.5 (48.6, 71.6)	58.5 (42.1, 73.7)	22 (14.3, 31.4)	37.5 (18.8, 59.4)
**Epicardial (%, CI)**	0 (0, 5.2)	2.6 (0.3, 9.2)	0 (0, 7)	17 (10.2, 25.8)	12.5 (2.7, 32.4)

Moreover, epicardial PCs were observed in almost 17% of the photomicrographs in the subacute reperfused infarction group. This finding was also present in 12% of the photomicrographs of the chronic reperfused infarction group. In comparison to the control group, no statistically significant differences in PC distribution were observed in the acute non-reperfused infarction group.

Differences were also observed regarding the perivascular distribution of PCs. No perivascular PCs were identified in the control group (0% (0–5.2)). Nevertheless, a significant amount of fibers appeared surrounding both arterial and venous coronary vessels in the infarction samples (11.8% (8–16.6); p<0.01).

The presence of perivascular PCs reached a maximum in the subacute reperfused infarction group, with approximately 20% of the micrographs exhibiting PCs with a perivascular pattern. This presence was significantly greater in this group than the control group (p<0.01). There were no differences between the subacute and chronic stages of reperfusion-infarction or between the control group and the acute non-reperfused infarction group (Table 1).

The frequency of perivascular PCs was also strongly correlated to the epicardial location, since 60.7% of the perivascular PCs were found in the epicardium (p<0.0001, Fisher exact test). No perivascular PCs were observed at endocardial level.

### 3. Other histological findings

In the acute reperfused infarction micrographs we noted patchy eosinophilia in the myocardial tissue. Coronary vessels appeared highly congested as a sign of microvascular obstruction (Fig 3C). In the non-reperfused infarction group, the findings were similar but microvascular obstruction was less frequent and predominantly arterial. After one week of reperfusion, coagulative necrosis with frequent contraction bands was found. The syncytium appeared highly disrupted, with a dense and mixed inflammatory infiltration composed of neutrophils, mononuclear cells and a remarkable number of eosinophils which in many regions had completely displaced the myocardial tissue. In some cases, PC bundles delimited areas with a preserved structure and others exhibiting intense degeneration (Fig 2B and 2C). In the chronic infarction model, fibrous scarring and inflammatory tissue containing small clusters of cardiomyocytes predominated.

## Discussion

The main findings of this experimental study can be summarized as follows: (1) Purkinje cells are able to survive after 90 minutes of myocardial ischemia with subsequent reperfusion. This initial number of surviving cells is present from the acute/sub-acute phase of reperfused infarction and remains unchanged until the chronic infarction stage. (2) The location of the PCs varies in the different stages of MI when comparing to control hearts. Purkinje cells reallocated from the mesocardium towards the endocardium and were distributed in close relation to perivascular areas within the pericardium, which may explain the possibility of catheter ablation of the re-entrant circuits of VT in chronic reperfused infarction.

Purkinje cells are known to be more resistant to ischemia than myocardial cells, as has been evidenced in canine hearts [1]. These cells are suspected to participate in ventricular arrhythmias after MI. The reported mechanisms are multiple: (1) Altered PCs may trigger electrical activity that induces ventricular ectopic beats or ventricular fibrillation in the acute phase of myocardial infarction [4, 14–18]; or (2) Purkinje cells may form part of a re-entry circuit in patients with post-infarction monomorphic ventricular tachycardia, more frequently in the chronic stages of infarction [2].

Various explanations of why human PCs are able to resist ischemia have been proposed. Subendocardial regions are better nourished by either sinusoidal channels or direct oxygen diffusion from the ventricular cavity. This might selectively condition greater survival of the subendocardial Purkinje network [1,19, 20]. Furthermore, PCs have been shown to be intrinsically more tolerant to hypoxia [21, 22]. In our study we also observed the ability of these cells to redistribute towards perivascular areas, such as those that remain preserved in the epicardium, after ischemia and reperfusion. This “migration” or new distribution could take place in the time between the first minute after reperfusion until one week later. After one week of reperfusion, no further changes in distribution are found. To our knowledge, this is the first study to describe this type of “migratory” sequence or anatomical redistribution during the subacute phase of reperfused infarction in pigs.

The presence of PCs mainly in the endocardium, and their capacity to survive up to the chronic phases of infarction lead us to suggest that they often participate in re-entrant arrhythmogenic circuits sustaining monomorphic ventricular tachycardias months or even years after MI.

Accordingly, PCs have the possibility of adopting a new distribution in the scar towards more oxygen-rich vascularized zones (present in the endocardium and perivascular zones in the epicardium) in the first week after infarction. This raises several hypotheses and suggests a number of future lines of research:

1. The first week after reperfused MI is decisive for the final location and surviving number of PCs, because no further displacements were observed in the chronic stage. Future treatments could be directed to avoid these changes in the scar in the first week.
2. Purkinje cells can appear in perfused peripheral zones such as the epicardium and endocardium, favoring future re-entrant circuits in these zones. A previous study by our group [23] also described that collagen fibers are homogeneously organized in the core of the infarction zone, and are randomly distributed in the endocardium, epicardium and peripheral area in chronic MI. Purkinje cells exhibit the same distribution. This special allocation (endocardium/epicardium) explains the possibility of catheter ablation of the re-entrant circuits most frequently from the endocardium [24] but also often from the epicardium [3, 24]. Participation in the origin of monomorphic ventricular tachyarrhythmias of the infarction core in a chronic a scar, which is inaccessible to catheter ablation, is fortunately less common.
3. Thus far there is no evidence regarding damage-induced differentiation or re-structuring of PCs in the adult heart in either humans or in other species. These results present an interesting basis for future molecular studies of previous signaling pathways and novel molecular factors.

The mechanism by which this new distribution of PCs is generated remains unknown. Migration or trans-differentiation from other types of cells after ischemic insult has been suggested [25–27]. Such redistribution could also be explained by extensive remodeling of the left ventricle itself. The thinning of the ventricular wall, cardiomyocyte necrosis, and the posterior desmoplastic response, could lead to the apparent relocalization of PCs from a central to a more peripheral position.

Several studies have addressed the histological changes of PCs in the acute phase of myocardial infarction. However, there is almost no information on the subacute and chronic stages of MI. Reversible subcellular alterations, mainly in subendocardial PCs, such as cytoplasm vacuolization, contraction bands or alterations in gap-junctional proteins, have been reported [1, 4–8, 11]. In our study we have corroborated the presence of lipid micro- and macrovacuoles, as they appear unstained after xylene processing, which selectively removes the lipid residues of the samples. Purkinje cell vacuolization predominates in the acute and subacute stages of MI, and exhibits a declining trend over time, decreasing notably in chronic reperfused infarction one month after ischemia. Moreover, a large number of vacuolated PCs are observed after reperfusion, what might be related to reperfusion-related damage [28]. These surviving PCs have been associated to altered action potentials and arrhythmogenic activity. In the acute phase, they exhibit reduced maximum diastolic potential, amplitude and depolarization velocity, directly related to the histological presence of cytoplasmic vacuoles [11]. This fact may play a role in the origin of ventricular fibrillation in the acute and subacute settings, but not in chronic monomorphic VT.

Finally, many studies have focused on the arrhythmogenic electrical properties of PCs in the acute phase of infarction [1, 11, 29–31]. We did not evaluate the arrhythmogenic capability of the preserved PCs in the different stages. However, we did confirm their easiness to survive after ischemia and to chronic stages of infarction, and their tropism towards vascularized areas in the chronic scar (i.e., endocardium and epicardium). In both regions, reentrant circuits have been described in clinical catheter ablation studies, establishing a possible underlying histological basis and pointing to the potential role of PCs in chronic stages.

## Conclusions

We have presented the first quantitative analysis of the distal cardiac conduction system in a porcine model representative of the different phases of MI, and the first reported change in the spatial distribution of PCs after MI. Purkinje cells are able to survive after 90 minutes of ischemia and subsequent reperfusion to a greater extent than cardiomyocytes. There is a decrease in the number of PCs, which suffer reversible subcellular alterations such as cytoplasmic vacuolization, as well as a redistribution of these cells from the mesocardium towards the endocardium and perivascular epicardial areas. However, these changes mainly occur during the first week after ischemia and reperfusion, and are maintained in the chronic stages. This anatomical substrate can explain the effectiveness of endo-epicardial catheter ablation of monomorphic VT in the chronic scar after infarctions, and sets the basis for further electrophysiological and molecular studies, and future therapeutic strategies.

## Study limitations

This study has several important limitations. Firstly, the sample size makes it necessary to view the results with caution and to carefully interpret the data, since the overall variability of the parameters could have been underrated, missing qualitative aspects such as the presence or absence of perivascular PCs in the anterior territory irrigated by the left descending coronary artery. Further information is needed in this regard.

As we have stated elsewhere, the number of PMJs could also be underestimated owing to tissue retraction during fixation processes, and their evaluation could be inaccurate. Electron microscopy would be the gold standard for determining truly coupled PMJs, but its use in this study seeking to cover large tissue areas was not feasible.

On the other hand, the parameter “Purkinje cell density” measures the percentage of tissue area occupied by PCs and not the actual number of cells, because the syncytial appearance of the conduction bundles usually precludes the counting of individual cells. Hence, density is influenced by survival of the surrounding tissue, and the values are of low magnitude, what limits the statistical power.

## Supporting information

S1 File ARRIVE guidelines checklist. Completed checklist in accordance to the ARRIVE Guidelines.
(PDF)
